# Carbon capture and storage in low-carbon concrete using products derived from olivine

**DOI:** 10.1098/rsos.231645

**Published:** 2024-05-01

**Authors:** Barney Shanks, Caitlin Howe, Sam Draper, Hong Wong, Christopher Cheeseman

**Affiliations:** ^1^ Centre for Infrastructure Materials, Department of Civil and Environmental Engineering, Imperial College London, , London SW7 2AZ, UK

**Keywords:** carbon sequestration, low-carbon cement, olivine, amorphous silica, nesquehonite

## Abstract

A novel process is reported that produces amorphous silica and nesquehonite (MgCO_3_·3H_2_O) from the magnesium silicate mineral olivine ((Mg, Fe)_2_·SiO_4_). The amorphous silica forms a supplementary cementitious material for use in concrete. The formation of nesquehonite sequesters carbon making the overall process carbon negative. Nesquehonite can also be used to form low-carbon construction products such as bricks, blocks and boards. This article reports on key process optimization studies. The potential for amorphous precipitated silica derived from olivine to produce carbon-negative concrete is discussed.

## 1. Introduction

Atmospheric CO_2_ concentrations are higher than at any time during the last 2 million years. With the Intergovernmental Panel on Climate Change finding a near-linear relationship between anthropogenic CO_2_ emissions and global warming, there is an urgent need to limit human-caused climate change by transferring to scenarios with very low greenhouse gas emissions [1]. This can be achieved through the development of low-carbon processes coupled with carbon capture and storage or carbon dioxide removal technologies [2].

The capacity for CO_2_ removal through mineral weathering of natural silicate minerals, such as olivine ((Mg, Fe)_2_SiO_4_) into carbonate minerals, is enormous. However, despite the potential to store thousands of gigatonnes of CO_2_, the rate at which weathering mineralizes CO_2_ is slow and occurs over geological timescales [3]. Natural weathering of basaltic rocks, including olivine, consumes only 9.5 tonnes CO_2_ yr^−1^ for every square kilometre exposed [4]. This amounts to approximately 66 Mt CO_2_ yr^−1^ being removed globally, which is approximately 0.2% of annual CO_2_ emissions [5]. The rate of mineral weathering can be increased by thermally, chemically or mechanically activating olivine, or by contacting olivine with high-pressure, high-purity CO_2_ in mineral carbonation processes. A simple example of mineral carbonation to accelerate this natural sink for CO_2_ is by distributing powdered magnesium silicate rock with a high surface area onto beaches and agricultural land to promote enhanced weathering [6].

The production of magnesium carbonates as a carbon sink has significant advantages. However, the increase in the rate of carbon mineralization through enhanced weathering remains unclear [7]. Using high-temperature, high-pressure and high-purity CO_2_ can increase the rate of reaction between olivine and CO_2_ by orders of magnitude. However, these processes require significant energy, limiting their effectiveness [8]. Complete olivine conversion requires approximately 2 tonnes of rock to sequester 1 tonne of CO_2_ from the atmosphere [9]. The resulting 3 tonnes of material (approx. 0.8 tonne SiO_2_ and 2.2 tonnes MgCO_3_) have no use in existing supply chains and no associated economic value [6].

Using aqueous conditions for direct carbonation makes it possible to use CO_2_ as carbonate anions [10]. Using aqueous acidic species to attack the olivine structure increases the rate of reaction beyond solid/gas contact reactions [11]. However, olivine samples treated by direct aqueous carbonation have 50–55 wt% olivine remaining, despite exposure to high energy conditions (175°C, 120 bar) after 2 h [12]. While efforts have been made to separate direct carbonation products after processing, the co-precipitation of silica and magnesium carbonate makes separation difficult [13].

There is a need to develop processes that deliver separate, high-purity reaction products that have intrinsic value from accelerated mineral carbonation. For example, contacting olivine rock with an acid, other than carbonic, and carbonating the metal ion leachate separately has the potential to significantly increase the reaction rate, while completely disaggregating silica production from carbonation through selective precipitation [14]. Indirect carbonation can be completed at lower temperatures and pressures, owing to the increased activity of the Mg^2+^ ions. A major problem encountered with aqueous acidic leaching of Si-rich minerals is the formation of amorphous silica hydrogels [15]. The silica gel formed is a non-filterable solid [16], making separation of the metal ion leachate extremely difficult, negatively impacting recovery and reducing the yield of subsequent carbonation reactions [17]. Drying the gel produces an insoluble amorphous precipitated silica (APS) and soluble metal salts, which must be removed by washing. This results in dilute metal ion solutions, impacting process efficiencies. Alternative methods for leaching in a dry environment have been suggested, but the leaching efficiency for some metals is reduced by approximately 40% without sufficient water present [18].

This article reports on a novel olivine acid digestion process that has a high rate of reaction and low energy consumption. In addition, this article presents a novel procedure that separates the reactive amorphous silica from the Mg^2+^ ion leachate, which is subsequently carbonated to form hydrated magnesium carbonates (HMC), with high efficiency. The research has determined the viability and efficiency of acid dissolution, product separation and carbonation. The effects of acid concentration on dissolution yields and reaction times, and the conditions required to precipitate high-purity silica and carbonate products to maximize potential value in subsequent applications in the built environment are reported.

**Figure 1.**
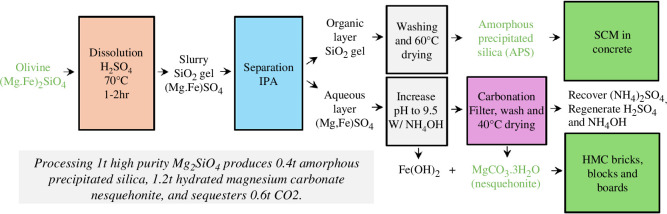
Schematic diagram showing the production of APS and HMC from olivine. The APS can be used as an SCM in concrete while the HMC sequesters carbon and has the potential for use in building products such as bricks, blocks and boards.

**Figure 2.**
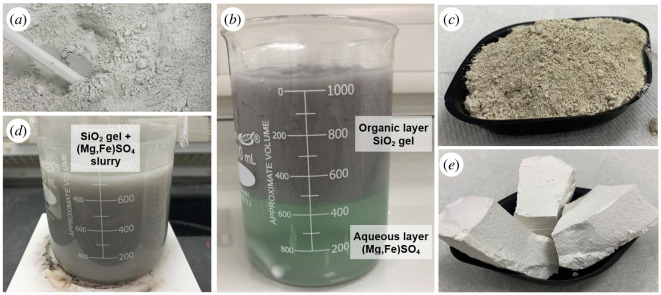
Photographs showing (*a*) raw as-received olivine, (*d*) dissolution of the olivine using H_2_SO_4_, (*b*) separation of silica gel from magnesium/iron sulphate using IPA, (*c*) APS and (*e*) HMC nesquehonite, produced from the process outlined in figure 1.

Figure 1 shows a schematic diagram of the process used to produce silica and nesquehonite (MgCO_3_·3H_2_O) from olivine [19]. This involves the dissolution of the olivine using sulphuric acid and the separation of the silica gel and magnesium/iron sulphate using isopropyl alcohol (IPA). The silica gel is then dried to form APS, and this can be used as a supplementary cementitious material (SCM) in concrete. Carbonation of the magnesium sulphate solution forms the HMC, nesquehonite, that can be used as a binder, filler or aggregate in other low-carbon construction products such as bricks, blocks and boards. Figure 2 shows photographs of the as-received olivine, acid dissolution of the olivine, separation of the silica gel using IPA, the APS and HMC, nesquehonite (MgCO_3_·3H_2_O) produced. The following sections describe the experiments completed to verify and optimize this process.

## 2. Experimental

### 2.1. Materials

**Figure 3.**
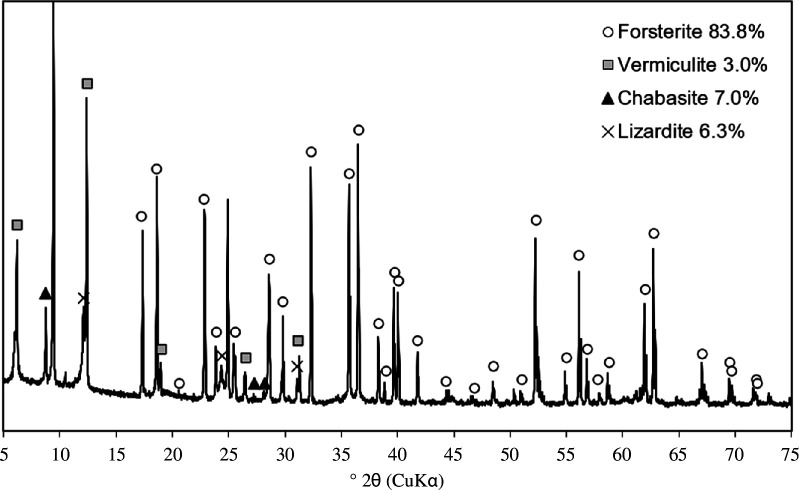
XRD diffractogram of the as-received olivine, indicating the presence of forsterite with minor amounts of chabazite, lizardite and vermiculite.

Olivine ((Mg, Fe)_2_ SiO_4_) was obtained from Sibelco Nordic AS (Aaheim Plant). X-ray diffraction (XRD) used a Malvern Panalytical Empyrean diffractometer over a scanning range from 5 to 75°2θ, with Ni-filtered CuKα radiation, at a scanning rate of 0.1°2θ s^−1^. Powder samples (approx. 1 g) were ground in an agate pestle and mortar (Cole-Parmer) and the less than 180 µm fraction separated by sieving was used in the XRD analysis. XRD data of the as-received olivine is shown in figure 3. This shows that the olivine contains more than 80% forsterite (Mg_2_SiO_4_), approximately 7% zeolite chabazite ((Ca,K_2_,Na_2_,Mg)Al_2_Si_4_O_12_·6H_2_O), approximately 6% lizardite (Mg_3_(Si_2_O_5_)(OH)_4_) and approximately 3% vermiculite ((Mg, Fe^2+^, Fe^3+^)_3_[(Al,Si)_4_O_10_]).

X-ray fluorescence (XRF) oxide composition data were obtained using a Malvern Panalytical Zetium. Samples were prepared as fused beads at 1050°C, with a lithium borate flux. Table 1 summarizes XRF composition data of the as-received olivine, expressed as oxides, showing that approximately 93% of metal ions (M^2+^) within the olivine are Mg with Fe being the other major cation present. The low content of Al indicates that vermiculite is a minor impurity.

**Table 1.** XRF oxide composition of as-received olivine, HMC derived from olivine and HMC synthesized from reagents.

**sample**	**composition (wt. %)**											
	SiO_2_	SO_3_	MgO	Fe_2_O_3_	CaO	Na_2_O	K_2_O	Al_2_O_3_	Cr_2_O_3_	NiO	other	total
olivine	39.80	0.85	48.71	6.76	0.18	0.83	0.08	0.87	0.44	0.36	0.19	99.07
HMC-olivine	0.37	7.24	81.65	7.78	0.32	—	—	0.14	—	—	0.49	98.00
HMC-synthetic	0.09	15.11	82.29	0.06	0.18	—	—	0.02	—	—	0.24	97.99

**Figure 4.**
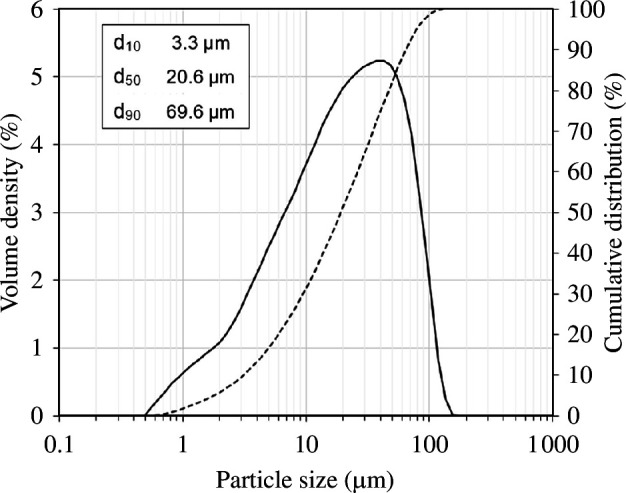
Particle size distribution data of the as-received olivine which has a *d*_50_ value of ~20 µm.

**Figure 5.**
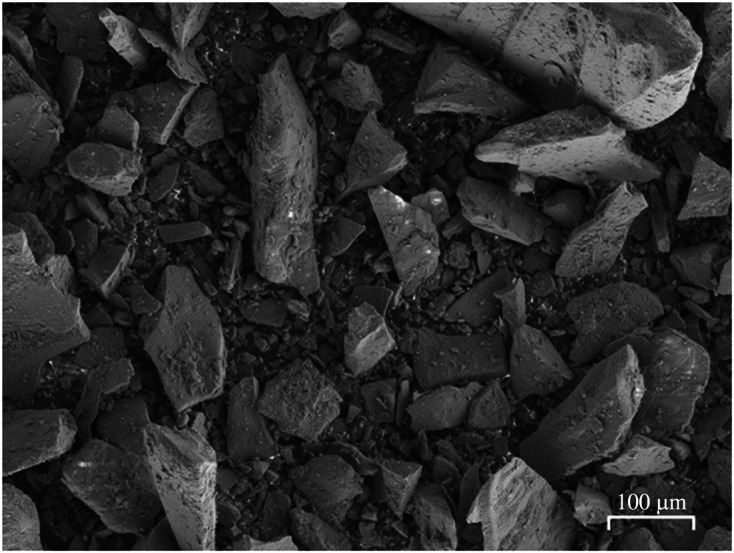
Backscattered scanning electron microscopy image of the as-received olivine.

The particle size distribution of the as-received olivine using laser diffraction granulometry (Malvern Mastersizer 3000) is given in figure 4. The particles have diameters (*d*) in the range of 0.5 < *d* < 200 µm with *d*_50_ value of approximately 20 µm. The backscattered scanning electron microscopy (SEM-BSE) image of the as-received olivine in figure 5 shows the angular nature of the olivine particles with particle sizes consistent with laser diffraction data.

Sulphuric acid (H_2_SO_4_, 96–98 wt%, Merrick), NH_4_OH (35 wt%, Sigma-Aldrich), MgSO_4_·7H_2_O (≥98 wt%, American Chemical Society, ACS reagent, Sigma-Aldrich) and IPA (99 wt%, Sigma-Aldrich) were used as chemical grade reagents. Erichrome® Black T (ACS reagent, indicator grade), ethanol (99.95 wt%, Sigma-Aldrich), MgCl_2_·6H_2_O (99.0–100.0 wt%, ACS reagent), NH_4_Cl (≥99.5 wt%, Sigma-Aldrich) and disodium ethylenediaminetetraacetic acid (EDTA) (≥99 wt%, ACS reagent, Arcos) were used in titration experiments.

Thermogravimetric analysis (TGA; Netzch STA 449 F5 Jupiter) of samples used a temperature range between 25 and 600°C, at a heating rate of 10°C min^−1^ under air, flowing at 50 ml min^−1^.

### 2.2. Dissolution

In these experiments, 500 ml of H_2_SO_4_ with concentrations of 1.0, 1.5, 2.0, 2.5 and 2.75 M were placed in a 1 l beaker on a hotplate with a Teflon-coated metal stirring bar. The acid solutions were prepared by dissolving concentrated H_2_SO_4_ in deionized water. The acid was heated to 70°C prior to the addition of olivine, which used a 10% M excess of olivine over reaction times up to 120 min. In the early stages of the reaction, the slurry contained aqueous magnesium and iron sulphates and dissolved silica monomers (silicic acid) as shown in equation (2.1):


(2.1) $${\left(Mg,Fe\right)}_{2}SiO_{4}+2H_{2}SO_{4}\to 2\left(Mg,Fe\right)SO_{4\left(aq\right)}+H_{4}SiO_{4\left(aq\right)}.$$

As the reaction progresses, SiO_2_ is precipitated as a suspended hydrogel through a condensation polymerization reaction as shown in equation (2.2):


(2.2) $$H_{4}SiO_{4\left(aq\right)}\to SiO_{2\left(gel\right)}+2H_{2}O.$$

The final slurry formed consisted of a mixture of silica gel, (Mg, Fe)·SO_4_ solution and some residual olivine (if dissolution is incomplete). For each initial acid concentration, reaction progression was monitored by extracting a sample of the slurry. These samples were filtered using a 22 µm syringe filter and the M^2+^ ion concentration was determined by EDTA–M^2+^ titration [14].

### 2.3. Separation

The silica gel was separated from the (Mg, Fe)·SO_4_ aqueous solution by adding IPA. Water and IPA are miscible at any composition under ambient temperatures and pressures. However, in a ternary system containing a salt, the mixture undergoes a salt-induced liquid–liquid phase separation. The solution separates into two layers. The upper layer is an organic-rich layer containing the non-polar silica gel in IPA that can be easily removed. The bottom layer is an aqueous-rich layer containing the dissolved (Mg, Fe)·SO_4_. The organic-rich silica layer was separated using a syringe and dried at 60°C to remove excess IPA. The APS was then washed in tap water to remove impurities, filtered and dried again at 60°C.

Preliminary experiments indicated that both chemical and physical factors influence the effectiveness of the separation process. The Mg^2+^ concentration and relative volume of IPA control the amount of aqueous phase, and therefore, Mg^2+^ concentration is present in the organic-rich layer at equilibrium. The silica gel network can also contain pockets of aqueous phase, transporting these into the organic-rich layer. To understand the influence of these factors, and optimize the separation process, two experiments were completed by examining the purity of the organic-rich layer and extracted silica, as follows:

1. a simplified system was investigated, by preparing magnesium sulphate solutions at a range of concentrations from reagent-grade epsomite (MgSO_4_·7H_2_O), with no APS present. The Mg^2+^ concentration in the solution, and quantity of IPA added were varied to establish the effects on Mg^2+^ distribution and layer volumes. Magnesium sulphate concentrations of 1.00, 1.50, 2.00, 2.50 and 2.75 M were prepared by dissolving epsomite in distilled water. Then, 10 ml samples were transferred to individual tubes and for each sample, IPA was added at volumetric ratios (IPA : MgSO_4_) of 0.5, 1.0 and 1.5. The volumes of the separated organic-rich and aqueous-rich layers were measured from volume markings on the centrifuge tubes, and the Mg^2+^ concentration in the organic-rich layer was determined by EDTA–M^2+^ titration; and
2. in addition, congruent IPA-induced separation experiments were performed on slurry samples produced through olivine dissolutions at different concentrations. The dissolution reactions were carried out as above. The samples were then cooled to room temperature and decanted into three 100 ml samples. For each sample, IPA was added at volumetric ratios (IPA : slurry) of 0.5, 1.0 and 1.5. The separated organic-rich layer was removed using a syringe, and dried at 60°C, without washing. XRF was used to establish the effect of the initial M^2+^ concentration in the slurry and IPA added on the purity of the extracted silica.

### 2.4. Carbonation

Removal of the organic-rich silica-containing layer produced a (Mg, Fe)·SO_4_ solution. The M^2+^ concentration was measured using EDTA–M^2+^ titration [14] and the solution was diluted accordingly to 0.5 M. Additionally, synthetic MgSO_4_ solutions of 0.4 and 0.5 M were prepared by dissolving reagent-grade epsomite (MgSO_4_·7H_2_O) in distilled water. Therefore, carbonation of the two sulfate solutions and the carbonate products could be compared to understand the effect of Fe impurities in the system.

Before carbonation, the pH of the (Mg, Fe) SO_4_ solution was increased to 9.5 using NH_4_OH. This causes the Fe^2+^ present in the separated slurry sample to precipitate as Fe(OH)_2_ (equation 2.3). The precipitation results in an initial M^2+^ (now solely Mg^2+^) concentration of approximately 0.45 M:


(2.3) $$FeSO_{4\left(aq\right)}+2NH_{4}OH_{\left(aq\right)}\to Fe{\left(OH\right)}_{2\left(ppt\right)}+{\left(NH_{4}\right)}_{2}SO_{4\left(aq\right)}.$$

An identical pH-controlled carbonation was performed on both MgSO_4_ solutions. CO_2_ gas (99.8%, British Oxygen Company, BOC) was bubbled through the solution with constant stirring at approximately 250 ml min^−1^. The pH was monitored using a pH meter and maintained at 9.5 ± 0.2 through regular additions of NH_4_OH solution. The reaction was monitored for 90 min, at which point the carbonate precipitate was separated by vacuum filtration, washed using tap water and dried at 40°C for 24 h. The carbonation reaction was monitored by extracting a sample at regular intervals, filtering out any precipitates and measuring the remaining Mg^2+^ concentration by EDTA–M^2+^ titration:


(2.4) $$MgSO_{4\left(aq\right)}+CO_{2\left(g\right)}+2NH_{4}OH_{\left(aq\right)}+2H_{2}O\to MgCO_{3}.3H_{2}O_{\left(ppt\right)}+{\left(NH_{4}\right)}_{2}SO_{4\left(aq\right)}.$$

## 3. Results

### 3.1. Dissolution

**Figure 6.**
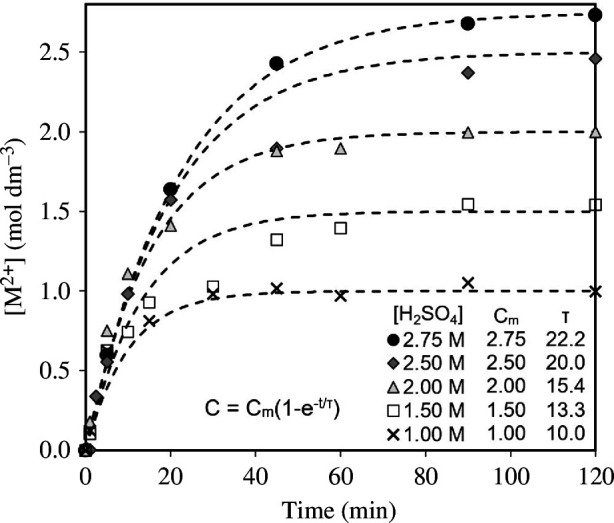
Concentration of M^2+^ ions in the slurry produced during olivine dissolution as a function of acid concentration and time. For each concentration, 10% M excess of olivine was used. All samples achieved more than 99% theoretical maximum dissolution.

Figure 6 shows the M^2+^ concentration in the slurry produced from the acid dissolution of olivine, as a function of H_2_SO_4_ concentration and time. The M^2+^ concentration is an indicator of the percentage of olivine dissolution. As expected, the rate of olivine dissolution is highest initially and decreases over time as reactants are consumed. The rate of dissolution increases with acid concentration.

The relationship between the concentration of M^2+^ ions in the slurry and the time elapsed for each initial acid concentration and olivine content can be represented by an exponential relationship *C* = *C*_m_(1 − e^-*t*/*τ*^) where *C* is the M^2+^ concentration at time *t*, *C*_m_ is the maximum theoretical concentration at complete dissolution and *τ* is a constant determined using regression analysis. This represents the rate of dissolution and is determined by the availability of reactants. The initial rates are high as reactants are present at high concentration. As the reaction progresses, reactants are consumed, and the reaction rate decreases.

**Figure 7.**
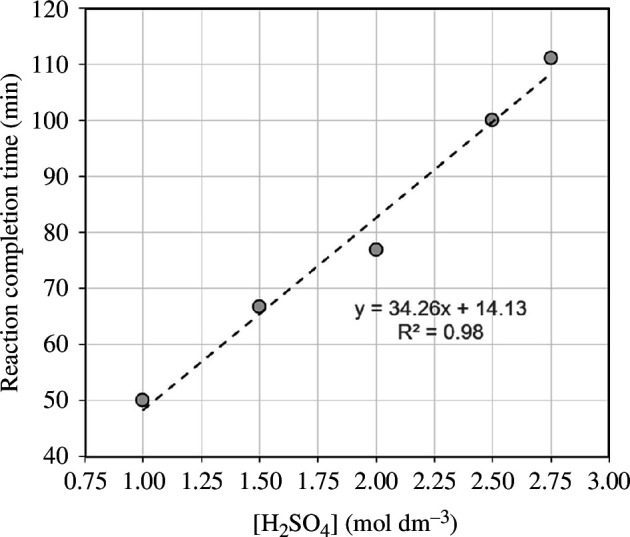
Relationship between initial acid concentration and reaction completion time, defined as the time at which 99% of the theoretical maximum dissolution *C*_m_ is achieved. The experiments used 10% M excess of olivine for each acid concentration.

Reaction completion time can be determined from the best-fit exponential equation as the time at which the concentration of dissolved M^2+^ ions is 99% of the theoretical maximum concentration *C*_m_. This corresponds to approximately 99% olivine dissolution. This occurs when time elapsed is equal to 5*τ*. At all initial acid concentrations, M^2+^ extraction reached over 99% of the theoretical maximum, therefore complete olivine dissolution is achieved. Figure 7 shows that the time to reach completion ranges from 50 to 110 min and is directly proportional to the initial acid concentration. The completion time increases with acid concentration because 10% M excess of olivine was used for each acid concentration.

### 3.2. Separation

**Figure 8.**
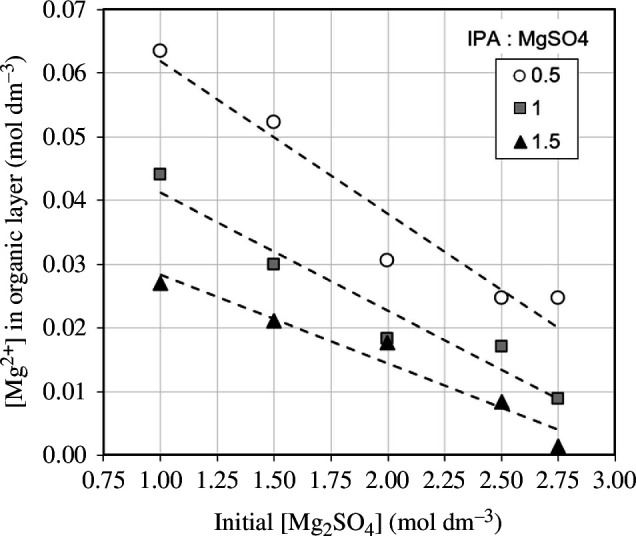
Influence of initial MgSO_4_ concentration and IPA : MgSO_4_ volume ratio in the simplified experiments on the concentration of Mg^2+^ in the organic-rich layer.

An efficient separation minimizes the amount of M^2+^ (Mg^2+^ only in simplified system) and SO_4_^2−^ in the organic-rich layer. Figure 8 presents data from these simplified separation experiments, showing how the initial MgSO_4_ concentration and added IPA volume impact the concentration of Mg^2+^ in the organic-rich layer, following phase separation. A strong inverse relationship was observed between the initial MgSO_4_ concentration and the concentration of Mg^2+^ in the organic-rich layer following the addition of IPA, consistent with previous work [20].

The solvation of Mg^2+^ and SO_4_^2−^ ions by water molecules is more energetically favourable than those same water molecules being dissolved in/solvating IPA. It is this preferential distribution of water molecules that causes the phase separation observed. However, there is a theoretical maximum amount of water that can be associated with the Mg^2+^/SO_4_^2−^ ions, which is determined by the size of the ions and their hydration spheres. The water that is not associated with these ions is ‘free’ to dissolve into the organic layer.

A lower initial Mg^2+^ concentration results in fewer Mg^2+^/SO_4_^2−^ ions present in the entire system for the same amount of water. Consequently, there is a greater amount of ‘free’ water, which is available to dissolve into the organic layer, increasing the volume of the organic-rich layer. This increases the solubility of the Mg^2+^/SO_4_^2^− ions in the organic layer, giving a greater proportion of these ions in the organic phase. Both an increase in volume and an increase in ions present in the organic layer having competing effects on the concentration of the organic layer. However, the increasing number of ions dominates giving an inverse relationship between initial [MgSO_4_] and [Mg^2+^] in the organic layer.

Additionally, a strong inverse relationship was observed between the volume of IPA addition and the concentration of Mg^2+^ in the organic-rich layer, following phase separation. The amount of IPA increases the separated organic-rich layer volume proportionally. This is owing to the preferential migration of organic solvent in the system into the organic-rich layer, consistent with previous research [21]. However, it has no measurable effect on the moles of Mg^2+^ in the organic-rich layer. As a result, as long as the critical volume of IPA is added to achieve phase separation, increasing this volume decreases the concentration of Mg^2+^ ions in the organic-rich layer.

In the IPA-induced separation experiments on slurries from olivine dissolution, reducing the amount of M^2+^ and SO_4_^2−^ in the organic-rich layer reduces the impurities (wt%) in the extracted silica after 60°C drying (unwashed). XRF data shown in table 2, indicates that the main impurities in the silica are Mg, Fe and SO_4_, which are likely to occur as MgSO_4_·xH_2_O, FeSO_4_·xH_2_O and residual olivine. The wt% impurities value has been calculated to include all species except silica, that is, as 100—wt%_SiO2_; this ranges from 25% to 85%.

**Table 2.** XRF oxide composition of silica samples (60°C dried, unwashed) extracted from olivine, showing the effect of initial M^2+^ concentration and IPA : slurry volume ratio on the purity of the silica.

**sample**		**c**omposition (wt.%)											
[M^2+^]	IPA : slurry	SiO_2_	SO_3_	MgO	Fe_2_O_3_	CaO	Na_2_O	K_2_O	Al_2_O_3_	Cr_2_O_3_	NiO	other	total
1.00	5	14.66	56.93	23.36	3.76	0.13	0.41	0.05	0.18	0.03	0.20	0.31	100.01
1.50	5	17.41	55.73	22.64	3.50	0.14	0.07	0.05	0.15	0.03	0.19	0.09	100.00
2.00	5	34.86	40.07	20.42	3.07	0.19	0.06	0.04	0.64	0.38	0.18	0.10	100.00
2.50	5	43.01	33.64	18.84	2.76	0.18	0.07	0.04	0.76	0.49	0.16	0.06	100.00
2.75	5	38.87	35.95	19.60	2.88	0.17	0.70	0.06	0.91	0.40	0.17	0.27	99.98
1.00	10	43.93	39.31	14.08	2.07	0.10	0.06	0.04	0.15	0.06	0.10	0.09	100.00
1.50	10	50.38	28.17	17.04	2.53	0.23	0.00	0.03	0.85	0.53	0.15	0.09	99.99
2.00	10	53.88	25.07	16.56	2.45	0.22	0.10	0.02	0.88	0.60	0.15	0.07	100.00
2.50	10	55.29	24.87	15.66	2.19	0.23	0.06	0.03	0.88	0.54	0.15	0.12	100.01
2.75	10	51.11	28.27	16.27	2.34	0.21	0.14	0.03	0.85	0.50	0.13	0.15	100.00
1.00	15	55.11	33.34	9.69	1.45	0.06	0.00	0.04	0.11	0.05	0.07	0.07	100.00
1.50	15	49.22	29.22	17.11	2.52	0.21	0.08	0.02	0.88	0.53	0.15	0.07	100.00
2.00	15	53.91	26.11	15.81	2.30	0.22	0.00	0.03	0.58	0.86	0.14	0.07	100.02
2.50	15	71.02	21.38	6.16	0.89	0.10	0.10	0.02	0.15	0.06	0.06	0.05	99.99
2.75	15	69.17	24.37	5.34	0.79	0.05	0.00	0.00	0.15	0.06	0.05	0.04	100.01

**Figure 9.**
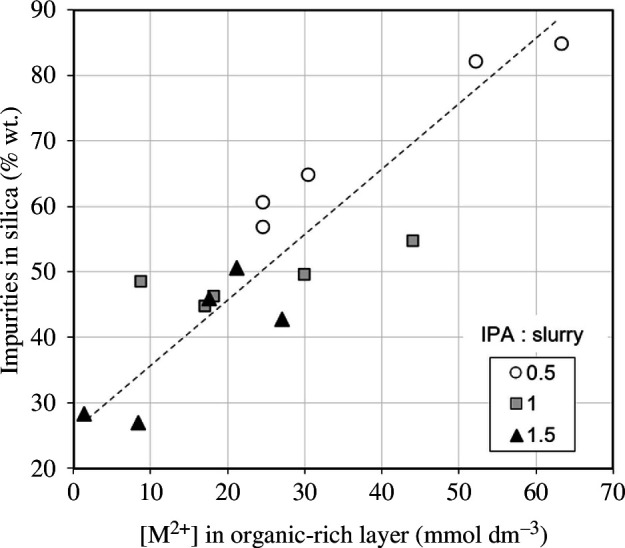
Influence of M^2+^ concentration in the organic-rich layer after olivine dissolution and separation on the impurities in the extracted silica (60°C dried, unwashed) at different IPA : slurry volume ratios.

Figure 9 shows that the impurity level of the extracted APS has a proportional relationship with the M^2+^ concentration in the organic-rich layer. This is probably a result of the combination of the two effects that comprise this concentration: the number of moles of ions in the organic layer and its volume. The absolute number of moles of ions impacts the impurities in the APS because dissolved species within the organic-rich layer are extracted along with the suspended silica gel network during separation.

The volume of the organic layer has been observed to physically impact the removal of impurities from the APS. For a given initial Mg^2+^ concentration, the amount of silica available after acid dissolution is constant, and the majority of this migrates to the organic-rich layer after IPA addition. A smaller organic-rich layer volume increases the solid/liquid ratio of the silica and organic-rich layer. As a result, the organic-rich layer becomes increasingly viscous, trapping a small proportion of the salt-rich, aqueous-rich layer, within the gel macrostructure. These salts then precipitate on drying, forming impurities within the APS. Therefore, the wt.% impurities in the extracted silica can be controlled and reduced by altering both the initial Mg^2+^ concentration and the volume of IPA added.

As shown by the simplified experiments, the concentration of Mg^2+^ in the organic layer is directly impacted by the initial MgSO_4_ concentration and the amount of IPA added. Therefore, minimizing the amount of Mg^2+^ in the organic-rich layer by increasing the initial MgSO_4_ concentration or increasing the addition of IPA presents a method for improving the purity of APS without requiring the need for extensive washing.

For a given magnesium concentration, the ‘best’ separation from an economic perspective involves adding the minimum amount of IPA that is sufficient for the silica gel network and this reduces the amount of salt carried into the organic-rich layer. The minimum amount of IPA must be chosen depending on the final application of the APS, to ensure that the wt% impurities is below any required threshold.

**Figure 10.**
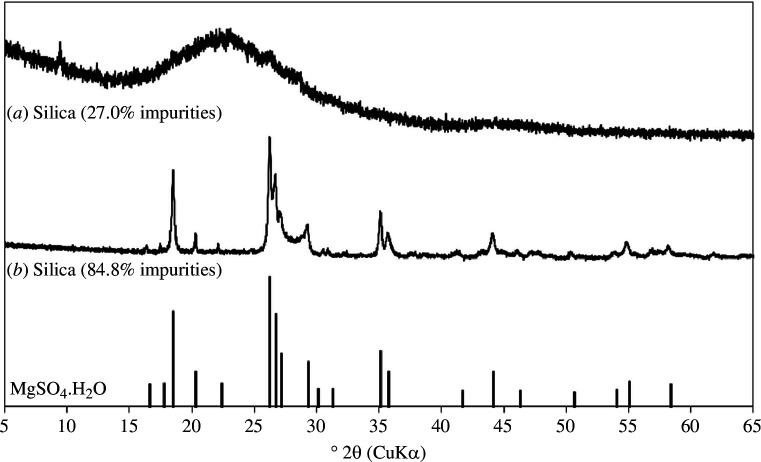
XRD diffractograms of extracted silica (60°C dried, unwashed) containing (*a*) 27.0% impurities showing an amorphous hump at around 22°2θ and (*b*) 84.8% impurities with peaks corresponding to MgSO_4_·H_2_O. Intensities are relative.

The XRD diffractogram of the extracted silica (60°C dried, unwashed) in figure 10 shows a large amorphous hump centred around 22°2θ in the case of high-purity samples. Therefore, a good separation process will yield highly reactive silica. Samples of lower purity show crystalline peaks characteristic of MgSO_4_·H_2_O. This indicates that the principal impurities within the silica recovered from the organic-rich layer are sulphate salts, predominantly MgSO_4_·H_2_O. As the silica gel produced is a non-filterable solid, these impurities cannot be removed easily by rinsing and the contaminated silica gel must first be dried before washing steps can be applied.

The water demand of the washing process can be calculated using equation (3.1), with the assumption that the soluble impurities are entirely MgSO_4_·H_2_O. Since the solubility of MgSO_4_·H_2_O in water is fixed, the amount of water required for washing is proportional to the impurity percentage estimated as:


(3.1) $$water\;demand\left(l/g,\;extracted\;silica\right)=\frac{\%impurities}{\%SiO_{2}}\times \frac{1}{solubility\;of\;impurities}.$$

**Figure 11.**
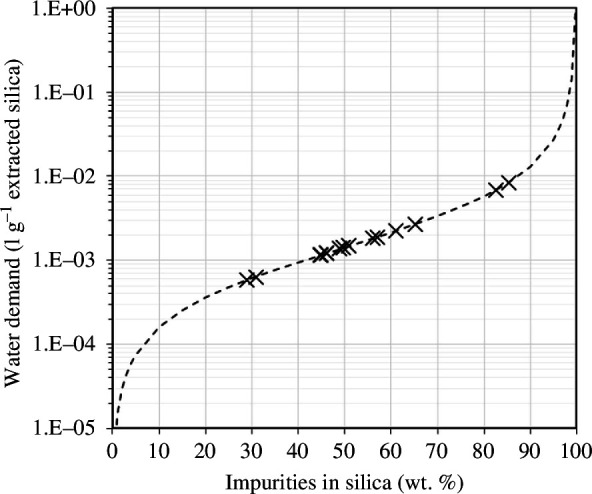
Estimated water demand required for washing to completely remove MgSO_4_·H_2_O impurities from the extracted silica.

The results are plotted in figure 11 for extracted silica with impurities content ranging from 27 wt% to 85 wt%. The estimated water demand required to completely purify these silica covers more than an order of magnitude, highlighting the importance of using efficient separation conditions. Overall, the results from this section show that high-purity silica (>70% SiO_2_) can be produced from optimized separation using IPA. With additional washing, a higher silica purity can be achieved.

### 3.3. Carbonation

**Figure 12.**
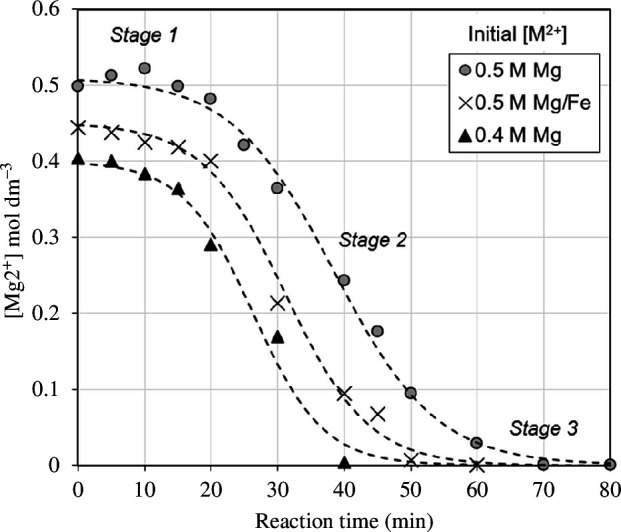
Change in Mg^2+^ concentration during carbonation of the synthetic MgSO_4_ solution (0.4 and 0.5 M), and the 0.5 M (Mg, Fe)·SO_4_ solution from olivine dissolution showing a three-stage reaction.

Figure 12 shows that carbonation is a three-stage reaction in both synthetic MgSO_4_ and separated-slurry samples of (Mg, Fe)·SO_4_ from olivine dissolution. An initial incubation period (stage 1) of approximately 15 min was observed where the Mg^2+^ concentration remained relatively constant for all samples. This is likely because bicarbonate ion (HCO_3_^−^) saturation is required for magnesium carbonate precipitation. Once the dissolved HCO_3_^−^ ions reach the saturation limit, steady-state precipitation occurs (stage 2). The rate of Mg^2+^ removal is linear through this stage and the equilibrium produces a constant rate of precipitation with bicarbonate dissolution as the rate-limiting step.

The dissolution rate of CO_2_ gas into water, and therefore HCO_3_^−^ ion concentration, is highly dependent on the pH of the solution (equation 3.2). Increasing pH, with a maximum pH value of 10, may decrease the incubation period and increase the rate of the linear stage of precipitation by increasing the bicarbonate activity (equations 3.3 and 3.4. The rate of precipitation during this period has been calculated at 1.36 g l^−1^ min^−1^, corresponding to sequestering 0.44 g CO_2_ l^−1^ min^−1^:


(3.2) $$CO_{2\left(g\right)}+H_{2}O_{\left(l\right)}\to HCO_{3\left(aq\right)}^{-}+H_{\left(aq\right)}^{+},$$

(3.3) $$HCO_{3\left(aq\right)}^{-}+H_{\left(aq\right)}^{+}+NH_{4}OH_{\left(aq\right)}\to NH_{4\left(aq\right)}^{+}+CO_{3\left(aq\right)}^{2-}+H_{2}O_{\left(l\right)},$$

(3.4) $$Mg_{aq}^{2+}+SO_{4(aq)}^{2-}+2NH_{4(aq)}^{+}+CO_{3(aq)}^{2-}+2H_{2}O_{\left(l\right)}\to MgCO_{3}\cdot 3H_{2}O_{\left(s\right)}+(NH_{4}{)}_{2}SO_{4(aq)}.$$

Finally, a third stage in the carbonation process was observed. This is associated with a reduction in Mg^2+^ consumption rate. This highlights a transition point at which the concentration of Mg^2+^ ions are rate determining with respect to carbonate precipitation, rather than the concentration of HCO_3_^−^ ions. It is also worth noting that the presence of Fe impurities from olivine did not have a significant effect on all three stages of the carbonation reaction.

The XRF oxide compositions of the precipitates extracted from carbonation of olivine-derived (Mg, Fe)·SO_4_ and synthetic MgSO_4_ are shown in table 1. The presence of SO_3_ is caused by incomplete washing. The carbonates derived from olivine show a trace quantity of SiO_2_, indicating the efficiency of the separation process. It also shows a higher weight percentage of Fe_2_O_3_ than the synthetic carbonate derived from reagent-grade epsomite. As the carbonate dries, Fe(OH)_2_ formed during the pH swing prior to carbonation oxidizes into various ferrous oxides.

**Figure 13.**
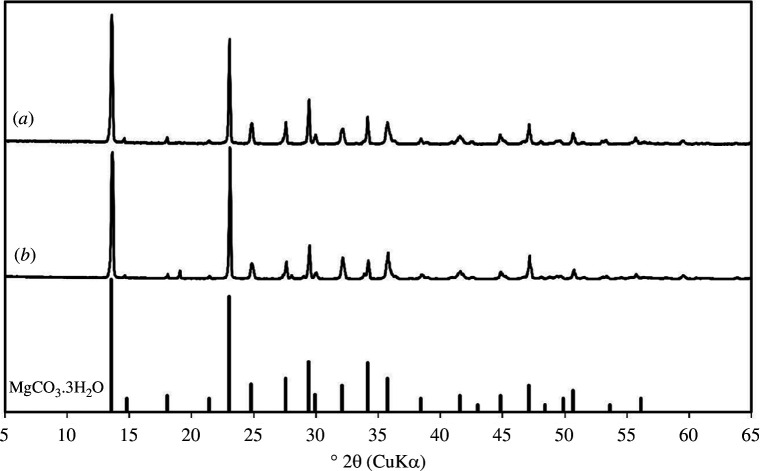
XRD diffractograms of precipitates from carbonation of (*a*) olivine derived (Mg, Fe)·SO_4_ and (*b*) synthetic MgSO_4_ solutions showing that the carbonates produced are mainly nesquehonite (MgCO_3_·3H_2_O).

XRD data presented in figure 13 indicate the carbonate precipitates formed is predominantly magnesium carbonate polymorph known as nesquehonite (MgCO_3_·3H_2_O). XRD diffractogram of carbonate precipitates from solutions containing Fe appear similar to those without it, suggesting that the impurities from olivine did not have a major influence on the products formed during the carbonation.

**Figure 14.**
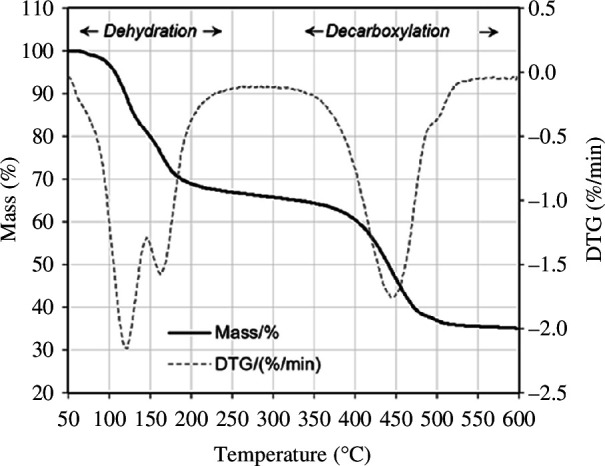
TGA and derivative thermogravimetry (DTG) data of precipitates from carbonation of olivine derived (Mg, Fe)·SO_4_ showing mass change consistent with that of nesquehonite.

Thermal analysis (figure 14) shows a two-stage mass loss of 34.3 wt% below 300°C, reflecting partial dehydration of nesquehonite, forming a phase with a lower content of crystal water, followed by total dehydration. This is slightly lower compared with the 39.0 wt% expected from the loss of three bound water molecules of nesquehonite. The difference can be attributed to impurities present in the sample. There may also be amorphous HMC phases present that are not identified by XRD, which contain less than three molecules of bound water. Above this temperature, the mass loss (30.4 wt%) is owing to decarboxylation and this is close to the theoretical value of 31.8%. Therefore, thermal activation and application of HMCs should be conducted below 300°C to ensure permanent storage of captured CO_2_.

**Figure 15.**
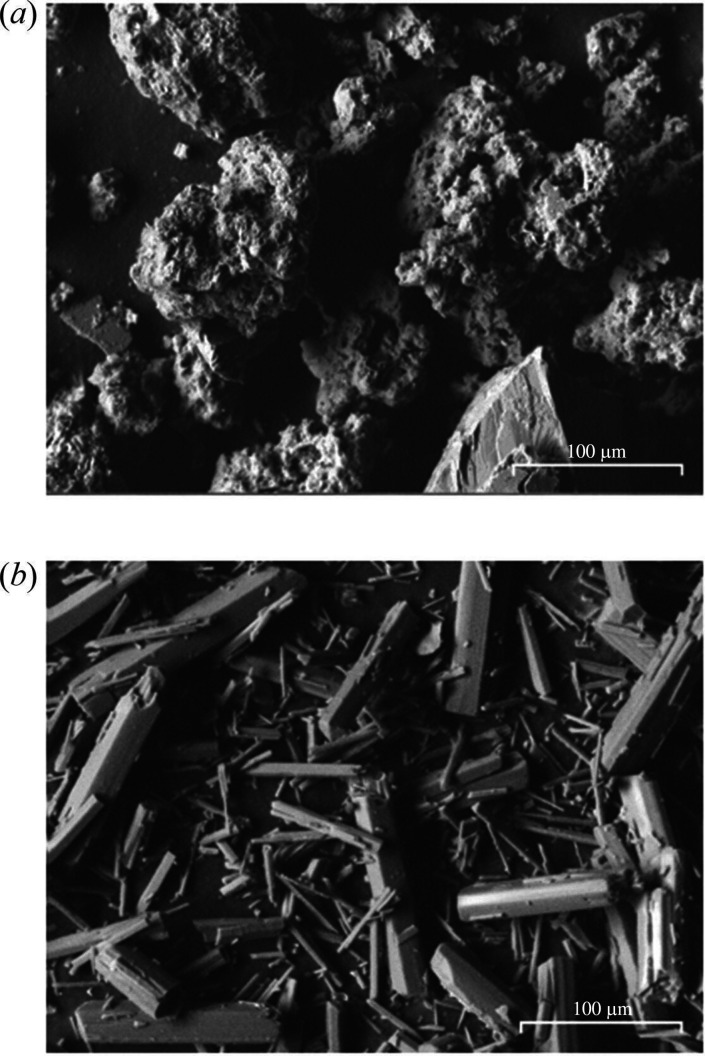
SEM-BSE image at 750× magnification of (*a*) APS and (*b*) nesquehonite derived from the process shown in figure 1.

Figure 15 shows SEM-BSE images of the APS and hydrated magnesium carbonate produced. The APS shows large agglomerates (approx. 100 mm) of fine micron-sized silica gel with rough microporous texture and high surface area. The HMC shows a needle-like structure of sub-100 mm in length that is characteristic of nesquehonite.

## 4. Discussion

The technology developed and optimized in this study involves a series of simple chemical processes that break down a globally abundant magnesium silicate mineral, olivine, to create two distinct products—amorphous silica and nesquehonite— with associated CO_2_ sequestration. Both products have multiple potential uses in construction materials and the process can use waste CO_2_ from industries such as cement production and power generation. The silica product from acid dissolution of olivine has an amorphous structure and this makes it highly reactive and valuable as an SCM that can be used to form carbon-negative cement and low-carbon concrete. The nesquehonite has the potential to be transformed into low-carbon blocks, bricks and boards. This is a low-cost chemical-based carbon capture process that generates two end products with commercial value as low-carbon construction materials. No other carbon capture and utilization systems processes currently provide these unique benefits.

**Figure 16.**
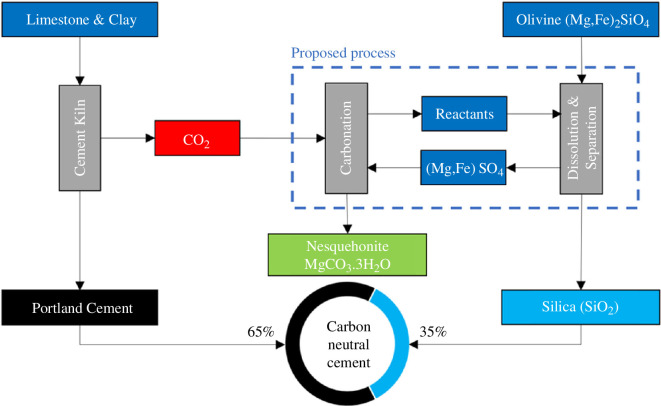
Schematic diagram showing how the processing outlined in this research could be used to sequester CO_2_ emitted from the production of PC. The CO_2_ is captured in nesquehonite to form low-carbon construction products. The silica is used to replace a fraction of PC in concrete. A carbon-negative composite cement is achievable at PC replacements greater than 35%.

Figure 16 shows a schematic diagram of how the process could be integrated with Portland cement (PC) manufacture to capture CO_2_ emissions and produce carbon-neutral PC. For 1 tonne of CO_2_ to be mineralized, 1–2 tonnes of olivine rock is required, accounting for the energy associated with mining, grinding and distribution [22]. When 1 tonne of high-purity *forsterite* (Mg_2_SiO_4_) is used for sequestration, equations (2.1)–(2.4) show that 0.4 tonne of silica and 1.2 tonnes of magnesium carbonate are generated, through the sequestration of 0.6 tonne of CO_2_. If the sequestered CO_2_ is assigned to the silica (eCO_2(min)_ = −1.47 tonnes eCO_2_ tonne^-1^), a carbon-neutral composite cement is achieved when 35% of the PC (eCO_2(max)_ = 0.80 tonne eCO_2_ tonne^-1^) is replaced with the silica SCM. Replacement levels greater than 35% could result in carbon-negative composite cements. It has been assumed that the olivine processing is not an energy-intensive process. Therefore, it can easily be electrified (ideally using renewables), incurring minimal additional emissions. However, the same assumption does not currently apply to cement manufacturing [23].

The cement industry releases between 2.5 and 3.0 Gt of CO_2_ yr^−1^, approximately 8% of global anthropogenic emissions [24]. Around 60% of this is chemically bound in the raw materials (mainly calcium carbonate) and released during the production of PC [25]. These process emissions are unavoidable and cannot be mitigated simply by improving efficiency or switching to clean energy. Concrete is central to the construction of buildings and infrastructure to sustain global prosperity. The current industry approach towards decarbonization is to use pozzolanic SCMs that are inherently lower in embodied carbon with PC to produce composite binders, to reduce raw material use, clinker factor and therefore the associated emissions. However, global supply of good quality conventional SCMs, such as coal fly ash and ground granulated blast-furnace slag, is in decline as industry shifts away from burning coal and recycled steel dominates production.

The European standard for cementitious materials (EN 197-1) [26] defines pozzolanic SCMs as containing a minimum of 25% reactive SiO_2_ by mass, with limits on other metal oxide, including MgO, and loss on ignition. By removing carbonate material and other impurities via an efficient separation process to increase the wt% of SiO_2_ (as shown in §4.2 and table 2), the olivine-derived silica product conforms well within industry standard. Therefore, the process described in this study can provide a new critical supply of reactive SCM and play a major role in the emission mitigation strategy of the PC/concrete industry, the construction sector and the built environment.

The process produces a large quantity of hydrated magnesium carbonate because this is where CO_2_ is stored. The HMC formed can also be used in a range of products capable of replacing conventional high-volume traditional construction materials that are widely used but carbon-intensive. Two important examples are clay-fired bricks and gypsum boards [27], which have significant environmental impacts and associated carbon emissions [28]. The setting and hardening mechanism of magnesium carbonate involves dehydration, through low-temperature thermal activation and rehydration of the carbonate material to re-form nesquehonite (MgCO_3_·3H_2_O), much like the hydration mechanism of anhydrous calcium sulphate into gypsum [29]. Another mechanism is via the phase transition of nesquehonite to hydromagnesite (Mg_5_(CO_3_)_4_(OH)_2_·4H_2_O), which can be accelerated at slightly elevated temperatures (approx. 60°C). Setting and hardening through these reactions means that a wide variety of construction products can be formed either by pressing, extrusion or casting. Other products may also be possible, such as magnesium carbonate as a filler to replace sand and aggregates in concrete production.

**Figure 17.**
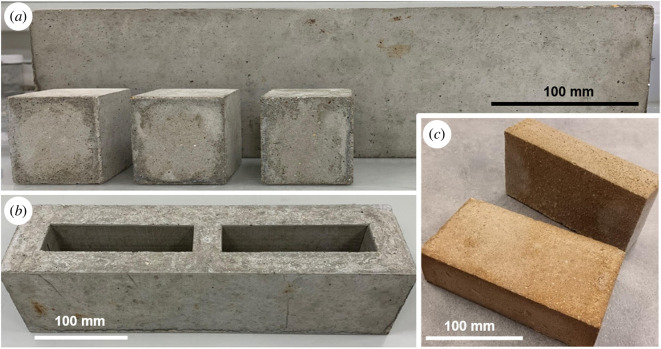
Example of carbon-neutral concrete blocks (PC : APS = 0.65 : 0.35, w/b ratio 0.5) (*a*,*b*) and magnesium carbonate bricks (HMC : sand = 1 : 6) (*c*), produced from the olivine-derived silica and nesquehonite described in this article.

Figure 17 shows an example of carbon-neutral concrete blocks and magnesium carbonate bricks produced from the olivine-derived silica and nesquehonite, respectively. Research has characterized the silica reactivity, and the setting and hardening mechanism of the magnesium carbonate at different temperatures, water/solid ratios and processing conditions. The silica has been tested in a range of concrete mixes, demonstrating comparable compressive strengths to typical SCMs such as fly ash at the same replacement level and age. Concretes with early-age (3 days) compressive strengths of 20–35 MPa and 28 days strength of 30–45 MPa have been achieved at 0.5 w/b ratio and 10–40% silica replacement. Using magnesium carbonate as the binder, we have produced bricks with strengths of 5–20 MPa by varying the w/b ratio, sand content and curing temperature/duration, exceeding industry performance requirements for many applications. The processing, properties and performance of these products will be reported in the following publications.

A limitation of the current study is the use of pure CO_2_ in the carbonation stage. Furthermore, the spent reagents are not recycled. Ongoing research includes establishing the effects of gas purity, flow rate, gas–liquid contact area, etc. and verifying the effectiveness of CO_2_ capture from industrial flues. Current research also includes optimizing a regeneration step to reduce waste and achieve circularity, and in doing so, reducing costs to ensure that the technology is viable and deployable at scale. Process flow modelling and complete life cycle assessment and life cycle cost analysis are also required. Further research to optimize the silica material and establish the long-term durability of concretes and stability of the magnesium carbonate products (and bound CO_2_) are ongoing.

## 5. Conclusions

A novel process for producing precipitated amorphous silica and nesquehonite, an HMC, from olivine, is reported. The silica has the potential to be used as an SCM in concrete and the production of nesquehonite sequesters carbon. As a result, when considering the chemical emissions, the silica is a carbon-negative SCM. The process involves acid dissolution using H_2_SO_4_ at 70°C and achieves complete extraction at a range of acid concentrations that were investigated. An efficient separation of dissolution products uses IPA. Impurities in the extracted silica and magnesium carbonates are impacted by the efficiency of this separation and can be controlled by altering process variables, such as maximizing acid concentration and using a sufficient IPA/slurry ratio of at least 1.5. High-purity silica (>70% SiO_2_) is produced from optimized separation, and this can be increased with additional washing steps. The presence of Fe in olivine has no significant effect on the rate of carbonation or the nesquehonite formed. The processing method reported has the potential to produce a carbon-negative silica SCM and nesquehonite that can be used in new low-carbon building materials.

## Data Availability

The datasets supporting this article are available online at Dryad [30].
